# Early removal of senescent cells protects retinal ganglion cells loss in experimental ocular hypertension

**DOI:** 10.1111/acel.13089

**Published:** 2019-12-22

**Authors:** Lorena Raquel Rocha, Viet Anh Nguyen Huu, Claudia Palomino La Torre, Qianlan Xu, Mary Jabari, Michal Krawczyk, Robert N. Weinreb, Dorota Skowronska‐Krawczyk

**Affiliations:** ^1^ Shiley Eye Institute Hamilton Glaucoma Center and Viterbi Family Department of Ophthalmology University of California, San Diego CA USA; ^2^ Richard C. Atkinson Lab for Regenerative Ophthalmology University of California, San Diego CA USA

**Keywords:** CNS, glaucoma, neuroprotection, senescence

## Abstract

Experimental ocular hypertension induces senescence of retinal ganglion cells (RGCs) that mimics events occurring in human glaucoma. Senescence‐related chromatin remodeling leads to profound transcriptional changes including the upregulation of a subset of genes that encode multiple proteins collectively referred to as the senescence‐associated secretory phenotype (SASP). Emerging evidence suggests that the presence of these proinflammatory and matrix‐degrading molecules has deleterious effects in a variety of tissues. In the current study, we demonstrated in a transgenic mouse model that early removal of senescent cells induced upon elevated intraocular pressure (IOP) protects unaffected RGCs from senescence and apoptosis. Visual evoked potential (VEP) analysis demonstrated that remaining RGCs are functional and that the treatment protected visual functions. Finally, removal of endogenous senescent retinal cells after IOP elevation by a treatment with senolytic drug dasatinib prevented loss of retinal functions and cellular structure. Senolytic drugs may have the potential to mitigate the deleterious impact of elevated IOP on RGC survival in glaucoma and other optic neuropathies.

## Graphical abstract

The authors show that early removal of senescent cells in the retina protects the healthy retinal ganglion cells from death to maintain vision. These data suggest the potential application of senolytic drugs to mitigate the deleterious impact of high intraocular pressure on retinal neurons.

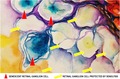

## 1 INTRODUCTION

Glaucoma is comprised of progressive optic neuropathies characterized by degeneration of retinal ganglion cells (RGC) and resulting changes in the optic nerve. It is a complex disease where multiple genetic and environmental factors interact (Skowronska‐Krawczyk et al., 2015; Weinreb, Aung, & Medeiros, 2014). Two of the leading risk factors, increased intraocular pressure (IOP) and age, are related to the extent and rate of RGC loss. Although lowering IOP is the only approved and effective treatment for slowing worsening of vision, many treated glaucoma patients continue to experience loss of vision and some eventually become blind. Several findings suggest that age‐related physiological tissue changes contribute significantly to neurodegenerative defects that cause result in the loss of vision.

Mammalian aging is a complex process where distinct molecular processes contribute to age‐related tissue dysfunction. It is notable that specific molecular processes underlying RGC damage in aging eyes are poorly understood. While no single defect defines aging, several lines of evidence suggest that activation of senescence is a vital contributor (He & Sharpless, 2017).

In a mouse model of glaucoma/ischemic stress, we reported the effects of *p16Ink4a* on RGC death (Skowronska‐Krawczyk et al., 2015). Upon increased IOP, the expression of *p16Ink4a* was elevated, and this led to enhanced senescence in RGCs and their death. Such changes most likely cause further RGC death and directly cause loss of vision. In addition, the analysis of p16KO mice suggested that lack of *p16Ink4a* gene protected RGCs from cell death caused by elevated IOP (Skowronska‐Krawczyk et al., 2015). Importantly, elevated expression of *p16INK4a* and senescence were both detected in human glaucomatous eyes (Skowronska‐Krawczyk et al., 2015). Therefore, for the first time, *p16Ink4a* was implicated as a downstream integrator of diverse signals causing RGC aging and death, both characteristics changes in the pathogenesis of glaucoma. Our findings were further supported by a subsequent report showing that *p16Ink4a* was upregulated by TANK binding kinase 1 (TBK1) a key regulator of neuroinflammation, immunity, and autophagy activity. TBK also caused RGC death in ischemic retina injury (Li, Zhao, & Zhang, 2017). Of particular note, a recent bioinformatic meta‐analysis of a published set of genes associated with primary open‐angle glaucoma (POAG) pointed at senescence and inflammation as key factors in RGC degeneration in glaucoma (Danford et al., 2017).

Glaucoma remains relatively asymptomatic until it is severe, and the number of affected individuals is much higher than the number diagnosed. Numerous clinical studies have shown that lowering IOP slows the disease progression (Boland et al., 2013; Sihota, Angmo, Ramaswamy, & Dada, 2018). However, RGC and optic nerve damage are not halted despite lowered IOP, and deterioration of vision progresses in most treated patients. This suggests the possibility that an independent damaging agent or process persists even after the original insult (elevated IOP) has been ameliorated.

We hypothesized that early removal of senescent RGCs that secrete senescent associated secretory proteins (SASP) could protect remaining RGCs from senescence and death induced by IOP elevation. To test this hypothesis, we used an established transgenic p16‐3MR mouse model (Demaria et al., 2014) in which the systemic administration of the small molecule ganciclovir (GCV) selectively kills *p16INK4a*‐expressing cells. We show that the early removal of *p16Ink4^+^* cells has a strong protective effect on RGC survival and visual function. We confirm the efficiency of the method by showing the reduced level of *p16INK4a* expression and lower number of senescent β‐galactosidase‐positive cells after GCV treatment. Finally, we show that treatment of p16‐3MR mice with a known senolytic drug (dasatinib) has a similar protective effect on RGCs as compared to GCV treatment in p16‐3MR mice.

## 2 MATERIALS AND METHODS

### 2.1 Animals

All animal experiments were approved by the UC San Diego Institutional Animal Care and Use Committee (IACUC) and adhered to the ARVO Statement for the Use of Animals in Ophthalmic and Vision Research. Adult p16‐3MR (Demaria et al., 2014) or C57BL/6 mice (12–16 weeks old, Jackson Labs) were housed in 20°C environment with standard (12 hr light/dark) cycling, food, and water available ad libitum. For all experiments, an equal number of male and female mice were used.

### 2.2 Drug treatment

**Figure 1:**
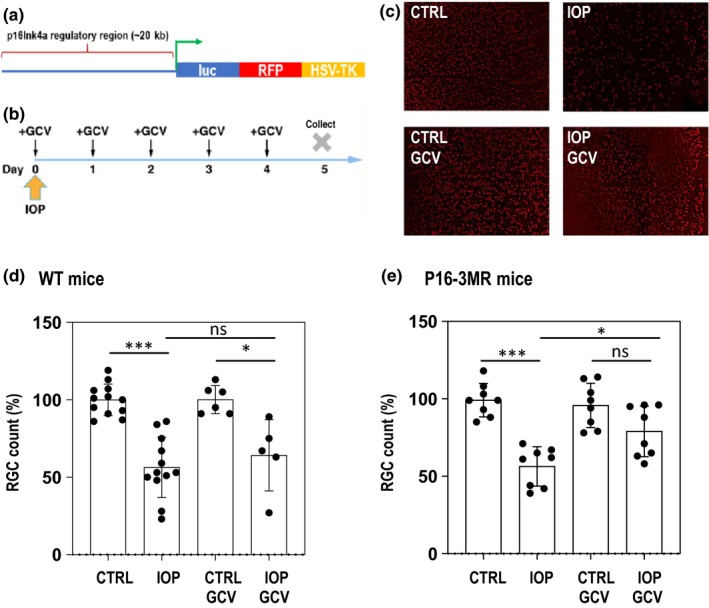
Removal of early senescent cells has a neuroprotective effect on RGCs. (a) Schematic representation of the p16‐3MR transgene. Triple fusion of luciferase, the red fluorescent protein, and tyrosine kinase from HSV virus are under control of the regulatory region of p16Ink4a gene. (b) Plan of the experiment. After unilateral IOP elevation mice are daily injected with GCV (25 mg/kg) intraperitoneally. At day 5 VEP is measured, and tissue is collected for further experiments. (c) Representative images of retina flat‐mount immunohistochemistry at day five with anti‐Brn3a antibody specifically labeling ~80% of RGC cells. (d) Quantification of RGC number at day five after the treatment of WT animals. *N* ≥ 5 animals in each group (e) Quantification of RGC number at day five after the treatment of p163MR animals. *N* = 8 animals in each group. In d and e, statistical tests were performed using ANOVA with post hoc Tukey correction for multiple testing. *p < .05, **p < .01, ***p < .001, n.s., not significant

The p16‐3MR transgenic model (Figure 1a), in which the mice carry a trimodal reporter protein (3MR) under the control of p16 regulatory region (Demaria et al., 2014), allows potent selective removal of senescent cells. The 3MR transgene encodes a fusion protein consisting of Renilla luciferase, a monomeric red fluorescent protein (mRFP) and herpes simplex virus thymidine kinase (HSV‐TK) which converts ganciclovir (GCV) into a toxic DNA chain terminator to selectively kill HSV‐TK expressing cells. The experimental group of animals was treated by intraperitoneal (IP) administration of GCV (Sigma, 25 mg/kg once a day) or dasatinib (Sigma, 5 mg/kg) after IOP elevation (see below), and a control group of mice was sham‐treated with PBS or vehicle (DMSO). Each mouse underwent unilateral hydrostatic pressure‐induced IOP elevation to 90 mm Hg, with the contralateral eye left as an untreated control. The mice were IP injected intraperitoneally with GCV or dasatinib at day 0 (IOP elevation day) and continued for four consecutive days (Figure 1b). At day 5, animals were euthanized, and retinas were isolated and immunostained with anti‐Brn3a antibody to evaluate the number of RGCs. All drugs were prepared according to the UC San Diego Institutional Animal Care and Use Committee (IACUC) standards. To ensure a sterile environment, compounds were prepared under the tissue culture hood using sterile PBS. The final solution was filtered through a 0.22‐µm PES membrane just before injection. Tips, tubes, and syringes were sterile.

### 2.3 Hydrostatic intraocular pressure (IOP) elevation

Animals were anesthetized with an intraperitoneal injection of ketamine/xylazine cocktail, (100 and 10 mg/kg, respectively), their eyes anesthetized with one drop of proparacaine (0.5%, Bausch‐Lomb) and dilated with one drop of tropicamide (1%, Alcon Laboratories). Unilateral elevation of IOP was achieved by infusing balanced salt solution (Alcon Laboratories) into the anterior chamber of the eye through using an intravenous (IV) infusion set. The level of IOP increase was determined by the height of the saline bottles on the IV infusion set. Stable elevated IOP of 85–90 mm Hg was maintained for 60 min and controlled by IOP measurements using a veterinary rebound tonometer (Tonovet). Both eyes were lubricated throughout testing with an ophthalmic lubricant gel (GenTeal, Alcon Laboratories). Animals recovered on a Deltaphase isothermal pad (Braintree Scientific) until awake. The contralateral eye without IOP elevation served as a healthy non‐IOP control (CTRL).

### 2.4 Visual evoked potential

VEP measurements were taken at five days post‐IOP elevation. This protocol was adapted from prior studies (Ridder & Nusinowitz, 2006). Mice were dark‐adapted for at least 12 hr before the procedure. Animals were anesthetized with ketamine/xylazine and their eyes dilated as above. The top of the mouse's head was cleaned with an antiseptic solution. A scalpel was used to incise the scalp skin, and a metal electrode was inserted into the primary visual cortex through the skull, 0.8 mm deep from the cranial surface, 2.3 mm lateral to the lambda. A platinum subdermal needle (Grass Telefactor) was inserted through the animal's mouth as a reference and through the tail as ground. The measurements commenced when the baseline waveform became stable, 10–15 s after attaching the electrodes. Flashes of light at 2 log cd.s/m^2^ were delivered through a full‐field Ganzfeld bowl at 2 Hz. Signal was amplified, digitally processed by the software (Veris Instruments), then exported, and peak‐to‐peak responses were analyzed in Excel (Microsoft). To isolate VEP of the measured eye from the crossed signal originating in the contralateral eye, a black aluminum foil eyepatch was placed over the eye not undergoing measurement. For each eye, peak‐to‐peak response amplitude of the major component P1‐N1 in IOP eyes was compared to that of their contralateral non‐IOP controls. Following the readings, the animals were euthanized, and their eyes collected and processed for immunohistological analysis.

### 2.5 Immunohistochemistry

Following euthanasia, eyes were enucleated and fixed in 4% paraformaldehyde (PFA) in PBS (Affymetrix) for 1 hr and subsequently transferred to PBS. The eyes were then dissected, the retinas flat‐mounted on microscope slides, and immunostained using a standard sandwich assay with anti‐Brn3a antibodies (Millipore, MAB1595) and secondary AlexaFluor 555 anti‐mouse (Invitrogen, A32727). Mounted samples (Fluoromount, Southern Biotech 0100‐01) were imaged in the fluorescent microscope at 20x magnification (Biorevo BZ‐X700, Keyence), focusing on the central retina surrounding the optic nerve. Overall damage and retina morphology were also taken into consideration for optimal assessment of the retina integrity. Micrographs were quantified using manufacturer software for Brn3a‐positive cells in 6 independent 350 × 350 µm areas per flat mount.

### 2.6 Real‐time PCR

Total RNA extraction from mouse tissues, cDNA synthesis, and RT‐qPCR experiments were performed as previously described (Skowronska‐Krawczyk et al., 2015). Assays were performed in triplicate. Relative mRNA levels were calculated by normalizing results using GAPDH. The primers used for RT‐qPCR are the same as in (Skowronska‐Krawczyk et al., 2015). The differences in quantitative PCR data were analyzed with an independent two‐sample *t* test.

### 2.7 SA‐βgal assay to test senescence on retinas mouse eyes

Senescence assays were performed using the Senescence b‐Galactosidase Staining Kit (Cell Signaling) according to the manufacturer's protocol. Images were acquired using a Hamamatsu Nanozoomer 2.0HT Slide Scanner and quantified in independent images of 0.1 mm^2^ covering the areas of interest using Keyence software.

### 2.8 RNA‐Seq analysis

High‐quality RNA was extracted using TRIzol Reagent (Invitrogen) and treated with TURBO DNA‐free Kit (Invitrogen). RNA‐Seq libraries were made from 1 µg total RNA per tissue sample using TruSeq stranded mRNA Library Prep Kit (Illumina, kit cat. no. 20020597) according to the manufacturer's instructions. The libraries were size‐selected using Agencourt Ampure XP beads (Beckman Coulter) and quality‐checked by Bioanalyzer (Agilent). The strand‐specific RNA‐Seq paired‐end reads sequence data (PE50: 2 × 50 bp) were obtained on a HiSeq4000. RNA‐Seq reads were counted using HOMER software considering only exonic regions for RefSeq genes.

## 3 RESULTS

Intraocular pressure was increased in one eye of transgenic mice bearing the p16‐3MR construct (Figure 1a). After IOP elevation, mice were intraperitoneally injected with GCV for five consecutive days (Figure 1b) to specifically deplete p16Ink4a‐positive (p16^+^) cells. In parallel, wild‐type animals were subjected to the same protocol, that is, underwent five daily GCV injections after unilateral IOP elevation. Retina flat‐mount immunohistochemistry and RGC quantification were used to assess potential impact of drug treatment. We observed that five‐day administration of GCV after IOP elevation had a significant protective effect on Brn3a^+^ RGC number in p16‐3MR mice when compared to untreated eye (Figure 1c,1). This protection was not observed in WT animals (Figure 1e) confirming that the effect of the GCV injection is specific to the mice harboring the GCV‐sensitive transgene.

**Figure 2:**
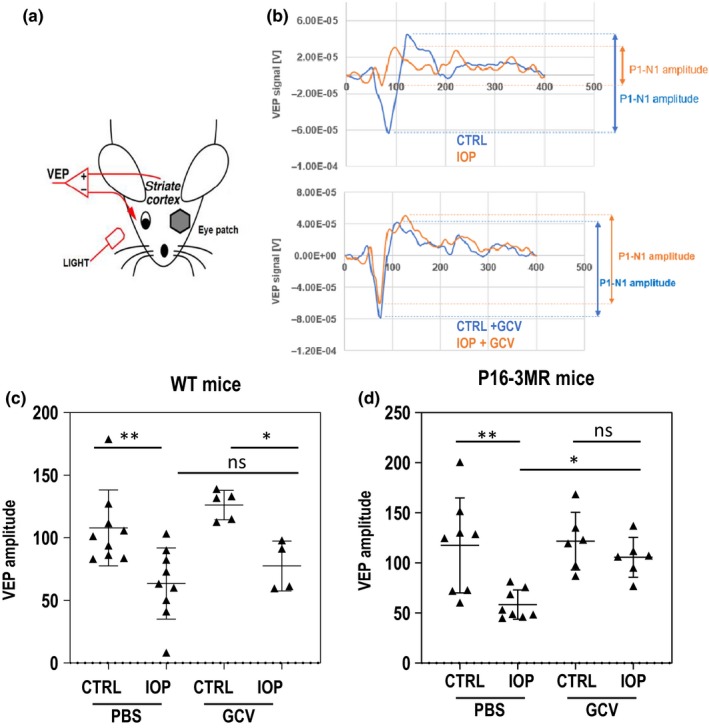
Visual functions are preserved in animals when senescent cells were removed. (a) Schematic representation of the placement of electrodes for VEP measurements. (b) Example results of VEP experiments. Top: results of the VEP response of healthy and IOP‐treated eyes. Bottom: After GCV injections, the VEP response is protected. (c) Quantification of VEP responses at day five after the treatment of WT animals. *N *≥ 4, (d) Quantification of VEP responses at day 5 after the treatment of the p16‐3MR animals. *N *≥ 6. In c and d, statistical tests were performed using ANOVA with post hoc Tukey correction for multiple testing. *p < .05, **p < .01, ***p < .001, n.s., not significant

Next, to test whether the protection of RGC numbers in GCV‐treated retinas was accompanied by the protection of the visual circuit integrity on day five, the in vivo signal transmission from the retina to the primary visual cortex was assessed by measuring visual evoked potentials (VEP) (Figure 2a)(Bui & Fortune, 2004; Porciatti, 2015). In brief, the reading electrode was placed in the striate visual cortex, with the reference electrode in the animal's mouth and ground electrode in the tail. Flash stimuli were presented in a Ganzfeld bowl. Response amplitudes were quantified from the peak‐to‐peak analysis of the first negative component N1. Using this approach, we have found that eyes subjected to IOP elevation showed decreased VEP P1‐N1 amplitude (Figure 2b), compared to contralateral non‐IOP control eyes. However, there was a marked rescue of VEP signals in transgenic animals treated with GCV (Figure 2b). Further quantification showed significant vision rescue upon GCV treatment only in p16‐3MR and not WT animals (Figure 2c,2), confirming the specificity of GCV treatment.

Our previous studies indicated that the increase in *p16INK4a* expression could be first observed as early as day three post‐IOP elevation (Skowronska‐Krawczyk et al., 2015). Therefore, we chose this time‐point to analyze the effectiveness of GCV treatment on senescent cells in treated and control retinas of p16‐3MR animals. RGC quantification showed that in animals not injected with GCV only ~15%–20% of cells disappeared at day 3 (compared to ~45%–50% on day 5).

**Figure 3:**
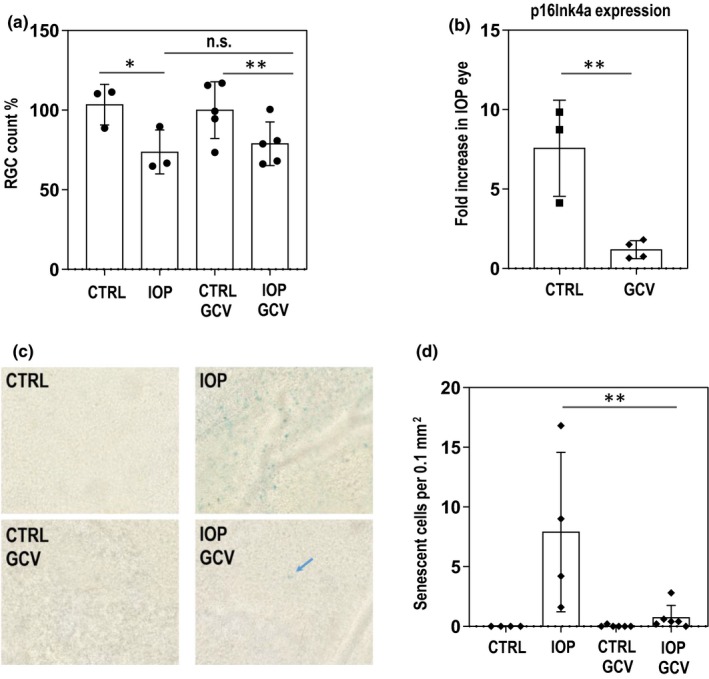
Senescence is lowered upon GCV treatment ~2 days before the effects on RGC numbers are observed. (a) At day 3 after IOP, only 20% of RGCs are lost compared to the non‐treated eye. Similar numbers of cells are lost in GCV‐treated eyes at this stage. *N* = 3 (non‐GCV) and *N* = 5 (GCV), ANOVA, *p < .05, **p < .01, n.s. – not significant (b) p16Ink4a expression is significantly lower in affected retinas isolated from GCV‐treated p16‐3MR animals at day 3 after IOP elevation. t‐test, **p < .01 (c) Number of SA‐b‐gal positive cells is lowered upon GCV treatment. Blue arrow – remaining senescent cell (d). Quantification of number of senescent cells upon IOP elevation in retinas isolated from mouse treated and non‐treated with GCV. *N* = 4 (non‐GCV), *N* = 6 (GCV); ANOVA, **p < .01

To test whether GCV treatment indeed removed senescent cells in the retina, we used two approaches. First, we quantified the *p16Ink4a* expression at day three post IOP treatment in GCV‐treated and control retinas. Expectedly, GCV treatment prevented IOP‐induced increase in *p16Ink4a* expression observed in non treated eyes (Figure 3b). Importantly, this was accompanied by significant decrease in numbers of IOP‐induced β‐galactosidase‐positive senescent cells in 3‐day GCV treated retinas as compared to nontreated cells (Figure 3c,3). This indicates that IOP‐induced early senescent cells are efficiently removed by GCV treatment by day 3, what precedes the RGC loss observed in non‐treated eyes between day 3 and day 5.

We next set forward on trying to understand molecular changes underlying the apparent protective effect of the removal of senescent cells by GCV treatment in p16‐3MR animals.

**Figure 4:**
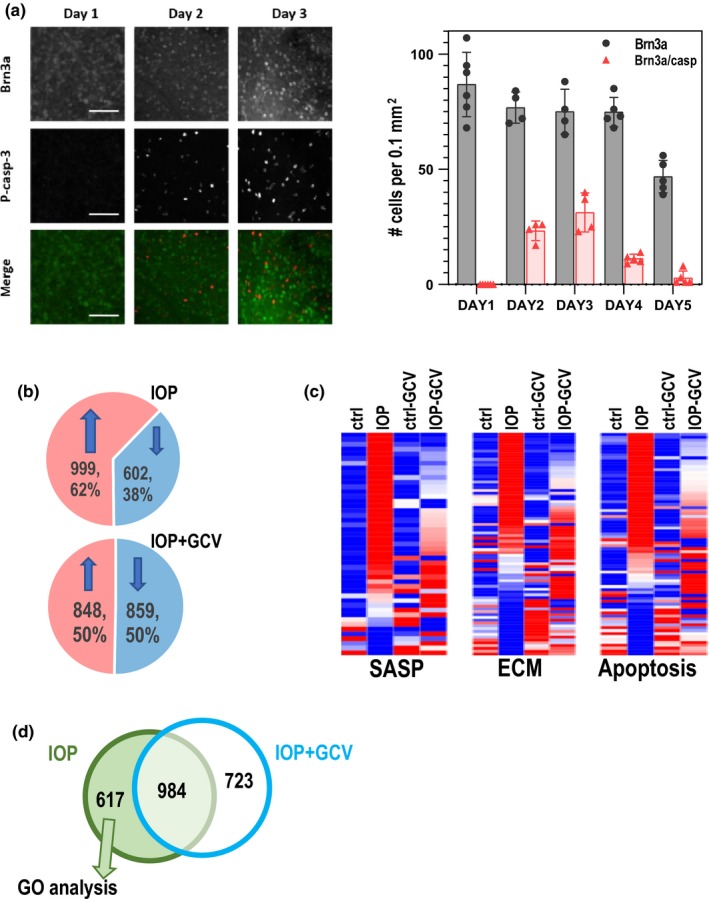
Analysis of pathways affected in IOP‐treated retinas. (a) Immunohistochemistry of Brn3a and activated caspase show increase of apoptosis at days1, 2 and 3 after IOP treatment. left: quantification of the time‐course experiments followed by immunochemistry with Brn3a and activated caspase 3; (b) RNA‐seq analysis of response to IOP and GCV. Eyes subjected to IOP elevation show significant change in gene expression with more genes upregulated than downregulated. Similar analysis in GCV‐treated animals shows close to equal distribution of upregulated and downregulated genes c. Heatmap analysis of genes involved in senescence, active oxidative species (ROS), apoptosis, extracellular matrix homeostasis (ECM), and inflammation. (d) Venn diagram showing overlap between genes dysregulated upon IOP in GCV‐treated and GCV‐untreated retinas. A total of 617 genes specifically dysregulated in IOP only retinas were used for GO analysis

First, we performed time‐course experiment in wild‐type mice to follow the activation of caspase expression as a marker of endogenous stress in the cell (Figure 4a). We quantified both total number of RGC (by nuclear staining for RGC specific transcription factor Brn3a) and activated caspase‐positive cells (by phospho‐caspase 3 staining). The highest number of RGCs with concomitant staining of activated caspase was observed at day 3 after IOP elevation (Figure 4a *right*). As shown above, at day 3 most of the RGCs are still present (Figure 3a). Day 3 also corresponds to the highest expression levels of senescence‐associated factors, as previously observed (Skowronska‐Krawczyk et al., 2015). We thus reasoned that relevant effects of GCV treatment should be easy to observe at around this stage. To identify potential differences in an unbiased way, we performed RNA‐seq analysis in IOP and non‐IOP retinas with or without GCV treatment. Total RNA isolated from 3 retinas in each experimental group was converted to cDNA libraries and sequenced. Of the total 21,351 detected gene loci, 1601 were significantly de‐regulated by the IOP treatment; 999 detected gene loci were up regulated; and 602 down‐regulated (Figure 4b, *top*). When the IOP treatment was performed in mice treated with GCV, the total numbers of IOP‐affected genes changed modestly to 1707, with 848 up regulated and 859 down‐regulated (Figure 4b, *bottom*).

To inquire in an unbiased way about the differences in signaling pathways and cellular processes affected by IOP, GO analysis using PANTHER was performed (Mi, Muruganujan, Ebert, Huang, & Thomas, 2019). This approach revealed that processes of the immune system response, inflammation, and extracellular matrix composition and cell–matrix interaction were significantly changed in IOP samples (Table 1). We have also detected the significantly deregulated genes involved in apoptosis, microglial activation and interlukin‐6 and interlukin‐8 production and secretion. This analysis shows that many mechanisms are induced upon an acute IOP elevation, most probably causing additional transcriptional stress to cell.

**Table 1:** List of pathways specifically deregulated in retinas upon IOP treatment

Analysis type: Annotation version and release date: Analyzed list: Reference list: Test type: Correction:	PANTHER Overrepresentation Test (Released 20190517) GO Ontology database Released 2019‐02‐02 upload_1 (Mus musculus) Mus musculus (all genes in database) FISHER FDR						
GO biological process complete	Mus musculus – REFLIST (22296)	Upload_1 (553)	Upload_1 (expected)	Upload_1 (over/under)	Upload_1 (fold Enrichment)	Upload_1 (raw P‐value)	Upload_1 (FDR)
Cellular response to interferon‐gamma (GO:0071346)	100	17	2.48	+	6.85	3.36E−09	2.94E−07
Positive regulation of interleukin−6 production (GO:0032755)	95	14	2.36	+	5.94	3.91E−07	2.40E−05
Protein ADP‐ribosylation (GO:0006471)	27	8	0.67	+	11.95	1.48E−06	7.66E−05
Positive regulation of gliogenesis (GO:0014015)	84	11	2.08	+	5.28	1.88E−05	7.29E−04
Lens development in camera‐type eye (GO:0002088)	69	10	1.71	+	5.84	2.05E−05	7.83E−04
Positive regulation of wound healing (GO:0090303)	55	9	1.36	+	6.6	2.25E−05	8.56E−04
Regulation of tyrosine phosphorylation of STAT protein (GO:0042509)	70	10	1.74	+	5.76	2.29E−05	8.70E−04
Response to interleukin−1 (GO:0070555)	87	11	2.16	+	5.1	2.53E−05	9.54E−04
Cellular response to interleukin‐1 (GO:0071347)	72	10	1.79	+	5.6	2.85E−05	1.06E−03
Positive regulation of superoxide anion generation (GO:0032930)	20	6	0.5	+	12.1	2.98E−05	1.10E−03
Integrin‐mediated signaling pathway (GO:0007229)	73	10	1.81	+	5.52	3.17E−05	1.16E−03
Respiratory burst (GO:0045730)	13	5	0.32	+	15.51	5.38E−05	1.90E−03
Negative regulation of cytokine‐mediated signaling pathway (GO:0001960)	48	8	1.19	+	6.72	5.72E−05	2.00E−03
Cellular response to cadmium ion (GO:0071276)	23	6	0.57	+	10.52	5.78E−05	2.01E−03
Negative regulation of interleukin‐6 production (GO:0032715)	49	8	1.22	+	6.58	6.52E−05	2.23E−03
Intrinsic apoptotic signaling pathway in response to DNA damage (GO:0008630)	81	10	2.01	+	4.98	7.05E−05	2.40E−03
Negative regulation of intracellular transport (GO:0032387)	66	9	1.64	+	5.5	8.11E−05	2.71E−03
Negative regulation of response to wounding (GO:1903035)	84	10	2.08	+	4.8	9.30E−05	3.05E−03
Lens fiber cell development (GO:0070307)	15	5	0.37	+	13.44	9.35E−05	3.06E−03
negative regulation of response to cytokine stimulus (GO:0060761)	52	8	1.29	+	6.2	9.47E−05	3.09E−03
Negative regulation of cell‐matrix adhesion (GO:0001953)	38	7	0.94	+	7.43	9.57E−05	3.11E−03
Synapse pruning (GO:0098883)	7	4	0.17	+	23.04	9.78E−05	3.16E−03
Protein poly‐ADP‐ribosylation (GO:0070212)	7	4	0.17	+	23.04	9.78E−05	3.16E−03
Lens fiber cell differentiation (GO:0070306)	26	6	0.64	+	9.3	1.04E−04	3.33E−03
Regulation of macrophage activation (GO:0043030)	53	8	1.31	+	6.09	1.07E−04	3.40E−03
Mononuclear cell migration (GO:0071674)	39	7	0.97	+	7.24	1.11E−04	3.51E−03
Regulation of homotypic cell–cell adhesion (GO:0034110)	29	6	0.72	+	8.34	1.75E−04	5.14E−03
Negative regulation of ERK1 and ERK2 cascade (GO:0070373)	75	9	1.86	+	4.84	1.96E−04	5.72E−03
Negative regulation of myeloid leukocyte mediated immunity (GO:0002887)	9	4	0.22	+	17.92	2.04E−04	5.89E−03
I‐kappaB kinase/NF‐kappaB signaling (GO:0007249)	45	7	1.12	+	6.27	2.44E−04	6.80E−03
Iron ion transport (GO:0006826)	45	7	1.12	+	6.27	2.44E−04	6.82E−03
Regulation of interleukin−8 production (GO:0032677)	61	8	1.51	+	5.29	2.56E−04	7.04E−03
Positive regulation of endothelial cell migration (GO:0010595)	97	10	2.41	+	4.16	2.73E−04	7.38E−03
Regulation of calcium‐mediated signaling (GO:0050848)	97	10	2.41	+	4.16	2.73E−04	7.39E−03
Protein mono‐ADP‐ribosylation (GO:0140289)	10	4	0.25	+	16.13	2.80E−04	7.52E−03
Positive regulation of neuron apoptotic process (GO:0043525)	79	9	1.96	+	4.59	2.79E−04	7.52E−03
Protein auto‐ADP‐ribosylation (GO:0070213)	11	4	0.27	+	14.66	3.75E−04	9.63E−03
Regulation of extrinsic apoptotic signaling pathway via death domain receptors (GO:1902041)	49	7	1.22	+	5.76	3.89E−04	9.93E−03
Protein transport within lipid bilayer (GO:0032594)	49	7	1.22	+	5.76	3.89E−04	9.95E−03
Acute inflammatory response (GO:0002526)	66	8	1.64	+	4.89	4.15E−04	1.06E−02
Positive regulation of hypersensitivity (GO:0002885)	12	4	0.3	+	13.44	4.90E−04	1.21E−02
Interleukin−6‐mediated signaling pathway (GO:0070102)	12	4	0.3	+	13.44	4.90E−04	1.22E−02
Negative regulation of wound healing (GO:0061045)	68	8	1.69	+	4.74	4.98E−04	1.23E−02
Regulation of interleukin−10 production (GO:0032653)	52	7	1.29	+	5.43	5.38E−04	1.31E−02
Negative regulation of receptor signaling pathway via JAK‐STAT (GO:0046426)	24	5	0.6	+	8.4	5.99E−04	1.42E−02
Microglial cell activation (GO:0001774)	24	5	0.6	+	8.4	5.99E−04	1.43E−02
Response to lipoprotein particle (GO:0055094)	24	5	0.6	+	8.4	5.99E−04	1.43E−02
Regulation of interleukin−12 production (GO:0032655)	53	7	1.31	+	5.33	5.96E−04	1.43E−02
Tolerance induction (GO:0002507)	13	4	0.32	+	12.41	6.29E−04	1.49E−02
Positive regulation of tyrosine phosphorylation of STAT protein (GO:0042531)	55	7	1.36	+	5.13	7.28E−04	1.69E−02
Cellular defense response (GO:0006968)	14	4	0.35	+	11.52	7.93E−04	1.82E−02
Cellular extravasation (GO:0045123)	40	6	0.99	+	6.05	8.05E−04	1.83E−02
Regulation of cell adhesion mediated by integrin (GO:0033628)	40	6	0.99	+	6.05	8.05E−04	1.84E−02
Cellular response to lipoprotein particle stimulus (GO:0071402)	26	5	0.64	+	7.75	8.23E−04	1.87E−02
Monoamine transport (GO:0015844)	26	5	0.64	+	7.75	8.23E−04	1.87E−02
Response to hydrogen peroxide (GO:0042542)	94	9	2.33	+	3.86	8.93E−04	1.99E−02
Positive regulation of membrane invagination (GO:1905155)	15	4	0.37	+	10.75	9.85E−04	2.16E−02
Regulation of hypersensitivity (GO:0002883)	15	4	0.37	+	10.75	9.85E−04	2.16E−02
Cellular response to low‐density lipoprotein particle stimulus (GO:0071404)	15	4	0.37	+	10.75	9.85E−04	2.17E−02
Positive regulation of vascular endothelial cell proliferation (GO:1905564)	15	4	0.37	+	10.75	9.85E−04	2.17E−02
Regulation of release of sequestered calcium ion into cytosol (GO:0051279)	79	8	1.96	+	4.08	1.22E−03	2.58E−02
Positive regulation of interleukin−6 secretion (GO:2000778)	29	5	0.72	+	6.95	1.27E−03	2.66E−02
Positive regulation of phosphatidylinositol 3‐kinase activity (GO:0043552)	29	5	0.72	+	6.95	1.27E−03	2.66E−02
Regulation of type I interferon production (GO:0032479)	80	8	1.98	+	4.03	1.32E−03	2.74E−02
Intrinsic apoptotic signaling pathway in response to DNA damage by p53 class mediator (GO:0042771)	31	5	0.77	+	6.5	1.65E−03	3.33E−02
Cellular response to amyloid‐beta (GO:1904646)	31	5	0.77	+	6.5	1.65E−03	3.33E−02
Toll‐like receptor signaling pathway (GO:0002224)	47	6	1.17	+	5.15	1.71E−03	3.42E−02
Inflammatory response to wounding (GO:0090594)	8	3	0.2	+	15.12	2.01E−03	3.89E−02
Positive regulation of cell adhesion mediated by integrin (GO:0033630)	19	4	0.47	+	8.49	2.08E−03	3.98E−02
Positive regulation of glial cell differentiation (GO:0045687)	49	6	1.22	+	4.94	2.08E−03	3.98E−02
Glial cell activation (GO:0061900)	33	5	0.82	+	6.11	2.12E−03	3.99E−02
Positive regulation of lipid kinase activity (GO:0090218)	33	5	0.82	+	6.11	2.12E−03	4.00E−02
Regulation of epithelial cell apoptotic process (GO:1904035)	87	8	2.16	+	3.71	2.15E−03	4.06E−02
Actin cytoskeleton reorganization (GO:0031532)	68	7	1.69	+	4.15	2.25E−03	4.21E−02
Response to axon injury (GO:0048678)	34	5	0.84	+	5.93	2.38E−03	4.41E−02
Regulation of oxidative stress‐induced neuron intrinsic apoptotic signaling pathway (GO:1903376)	9	3	0.22	+	13.44	2.64E−03	4.73E−02
Amyloid fibril formation (GO:1990000)	9	3	0.22	+	13.44	2.64E−03	4.76E−02
Hydrogen peroxide biosynthetic process (GO:0050665)	9	3	0.22	+	13.44	2.64E−03	4.76E−02
Response to amyloid‐beta (GO:1904645)	35	5	0.87	+	5.76	2.67E−03	4.76E−02
Neuroinflammatory response (GO:0150076)	35	5	0.87	+	5.76	2.67E−03	4.76E−02
Toll‐like receptor 3 signaling pathway (GO:0034138)	9	3	0.22	+	13.44	2.64E−03	4.77E−02
Actin‐myosin filament sliding (GO:0033275)	9	3	0.22	+	13.44	2.64E−03	4.77E−02
Positive regulation of acute inflammatory response (GO:0002675)	35	5	0.87	+	5.76	2.67E−03	4.77E−02

Further analysis revealed that the genes involved in cellular senescence, extracellular matrix molecules and in factors involved in apoptosis (Table 2) (Pawlikowski *et al.*, 2013) were significantly de regulated upon IOP elevation. Importantly, 3‐day treatment to remove p16 + cells significantly mitigated this response (Figure 4c). These data are in agreement with the loss of the senescence cells upon GCV treatment (Figure 3b3) and lower detrimental impact of senescent cells on surrounding cells.

**Table 2:** List of genes in pathways represented in Figure 4c

Apoptosis	ECM	SASP
Abl1	Adamts1	Ang5
Actb	Adamts2	Areg
Aifm1	Adamts5	Ccl11
Akt1	Adamts8	Ccl12
Anxa5	Cdh1	Ccl2
Apaf1	Cdh2	Ccl20
Api5	Cdh3	Ccl7
Atf5	Cdh4	Csf2
Bag1	Cntn1	Csf3
Bag3	Col1a1	Ctsb
Bak1	Col2a1	Cxcl11
Bax	Col3a1	Cxcl12
Bcl10	Col4a1	Cxcl13
Bcl2a1a	Col4a2	Egf
Bcl2l10	Col4a3	Egfr
Bcl2l2	Col5a1	Ereg
Bid	Col6a1	Fas
Birc2	Ctgf	Fgf2
Birc3	Ctnna1	Fgf7
Birc5	Ctnna2	Fn1
Bnip2	Ctnnb1	Hgf
Bok	Ecm1	Icam1
Card10	Emilin1	Ifng
Casp1	Entpd1	Igfbp2
Casp12	Fbln1	Igfbp3
Casp14	Fn1	Igfbp4
Casp2	Hapln1	Igfbp5
Casp3	Hc	Igfbp6
Casp4	Icam1	Igfbp7
Casp6	Itga2	Il13
Casp7	Itga3	Il15
Casp8	Itga4	Il1a
Casp9	Itga5	Il1b
Cd40lg	Itgae	Il6
Cd70	Itgal	Il6st
Cflar	Itgam	Il7
Cidea	Itgav	Mip
Cideb	Itgb1	Mmp12
Cradd	Itgb2	Mmp13
Dad1	Itgb3	Mmp14
Dapk1	Itgb4	Mmp1a
Dffa	Lama1	Mmp2
Dffb	Lama2	Mmp3
Diablo	Lama3	Mmp7
Fas	Lamb2	Mmp9
Fasl	Lamb3	Ngf
Gadd45a	Lamc1	Pigf
Gapdh	Mmp10	Plaur
Gusb	Mmp11	Serpinb2
Hsp90ab1	Mmp13	Serpine1
Igf1r	Mmp14	Timp1
Il10	Mmp15	Timp2
Lhx4	Mmp1a	Tnfrsf11b
Ltbr	Mmp2	Tnfrsf1a
Mapk1	Mmp3	Tnfrsf1b
Mcl1	Mmp7	Tnfrsf22
Naip1	Mmp8	
Naip2	Mmp9	
Nfkb1	Ncam1	
Nme5	Ncam2	
Nod1	Pecam1	
Nol3	Postn	
Polb	Sele	
Prdx2	Sell	
Ripk1	Selp	
Tnfrsf10b	Sgce	
Tnfrsf11b	Sparc	
Tnfrsf1a	Spock1	
Tnfsf10	Spp1	
Tnfsf12	Syt1	
Traf1	Tgfbi	
Traf2	Thbs1	
Trp53	Thbs2	
Trp53bp2	Thbs3	
Trp63	Timp1	
Trp73	Timp2	
Xiap	Timp3	
	Tnc	
	Vcam1	
	Vcan	
	Vtn	

**Figure 5:**
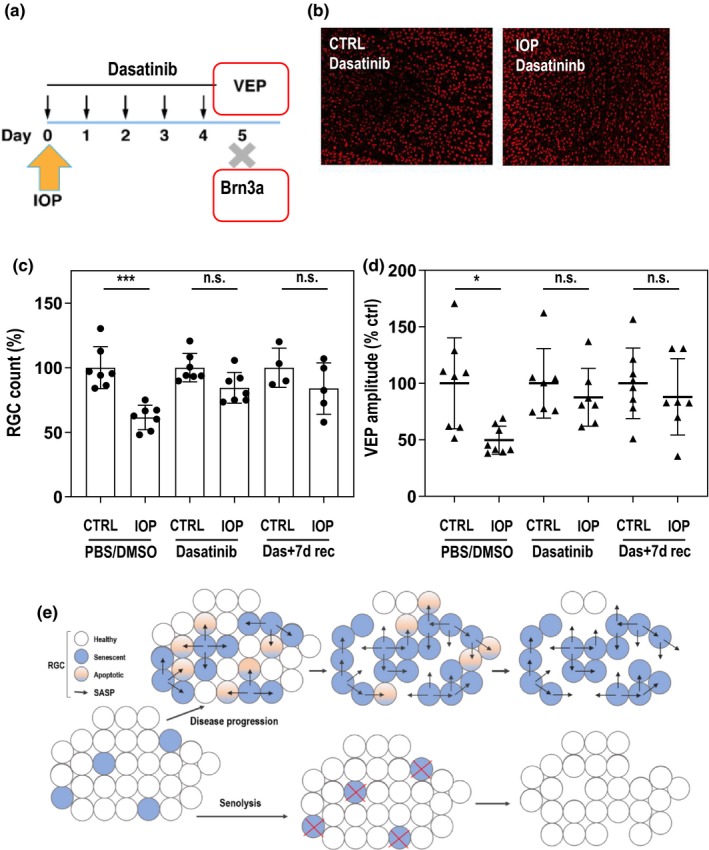
Dasatinib protects retina degeneration. (a) Plan of the experiment. After unilateral IOP elevation, mice are daily injected with dasatinib (5 mg/kg) intraperitoneally. At day 5, VEP is measured and tissue is collected for further experiments. Immunohistochemistry of Brn3a and activated caspase show increase of apoptosis at day 3 after IOP treatment. (b) Retina flat‐mount immunohistochemistry at day 5 with anti‐Brn3a antibody specifically labeling ~80% of RGC cells. (c,d) Quantification of RGC number (c) or VEP responses (d) at day 5 (four conditions) or day 12 (additional 7 days of “recovery,” two conditions) after the 5 days treatment of p16‐3MR animals with dasatinib. *N* > 4 animals in each group. Statistical tests were performed using ANOVA with post hoc Tukey correction for multiple testing. *p < .05, ***p < .001, n.s. – not significant (e). Model. Top: Upon elevated IOP damaged cells become senescent and start to express SASP molecules. While disease progresses, the SASP molecule induces senescence or apoptosis in neighboring cells. Bottom: When senescent cells are removed using senolytic drug the neighboring cells are not exposed to detrimental SASPs and the disease progression is significantly slowed down. Remaining cells are healthy

Additional GO analysis of the 617 genes which were significantly de regulated upon IOP elevation specifically in non treated retinas (i.e., genes where the effects of IOP were dampened by GCV‐mediated removal of senescent cells) (Figure 4d) identified a specific enrichment of a class of genes belonging to the ABL1 pathway and ABL1 downstream targets (Fig. S1). Prompted by this finding, we explored whether dasatinib, a well‐known senolytic drug and a Bcr‐Abl and Src family threonine kinase inhibitor, could have a beneficial effect similar to GCV in p16‐3MR mice. To this end, p16‐3MR mice were treated with dasatinib (5 mg/kg) or vehicle for 5 days by intraperitoneal injection, similarly to the experimental procedure used for GCV (Figure 1b). Performing this experiment in the transgenic mice allowed direct comparison of the efficiencies of both treatments in the same mouse strain. At day five after IOP elevation, VEP measurement was performed and retinas were immunostained to quantify RGC loss. We observed that dasatinib treatment prevented the loss of RGC (Figure 5c) similar to what was observed in GCV‐treated animals (Figure 1e). Most importantly, VEP analysis revealed that senolytic drug treatment successfully prevented vision loss upon IOP elevation (Figure 5d).

Finally, we explored whether the protective impact of the drug is caused by the sustained inhibition of the cellular processes and whether it is maintained even after the drug is no longer present. To do that, p16‐3MR mice were treated with dasatinib (5 mg/kg) or vehicle for 5 days by intraperitoneal injection, similarly to the experimental procedure used for GCV (Figure 1b). After that, the mice were no longer treated with drug or PBS and at day twelve after IOP elevation, functional measurement was performed and RGCs were quantified. Also in this treatment regime, dasatinib prevented the loss of RGC (Figure 5c) similar to what was observed in GCV‐treated animals (Figure 1e). Additionally, VEP analysis revealed that senolytic drug treatment with seven days “chase” still successfully prevented vision loss upon IOP elevation (Figure 5d).

## 4 DISCUSSION

The collective findings of the current study strongly support the notion that removal of senescent cells provides beneficial protective effect to retinas damaged by elevated IOP. Here, we show that in transgenic animals expressing viral TK under the control of regulatory regions of p16Ink4a (Demaria et al., 2014), selective removal of early senescent cells in the retina is beneficial for neighboring cells undergoing cellular stress induced by IOP elevation. In this model, the treatment with GCV selectively induces cell death of transgene‐expressing cells. Early application of GCV as soon as day 0 after the IOP elevation and followed for five consecutive days ensures early removal of *p16Ink4a* expressing cells resulting in protection of neighboring RGCs from death. Remaining cells are still able to provide a signal to the visual cortex, as evidenced by VEP measurements, demonstrating that protected cells are functional.

Three days after GCV injection, there is a significant drop in senescent cell number and an accompanying alteration of the transcriptional programs in remaining cells as compared to retinas from untreated mice. Using an RNA‐seq approach, we noted significant changes in the senescence program as well as in the extracellular matrix (ECM) function. Both pathways are downregulated by GCV treatment. Finally, we show that dasatinib, a known senolytic drug, can be used to protect RGCs from death, further confirming that early removal of senescent cells induced upon IOP elevation protects retina health. The observation also was confirmed when the RGC count and VEP were assessed seven days after the treatment was stopped; this suggests that the impact of the drug is not reversible during this time. Further studies should investigate different regimes and dosages of the senolytic drugs and their neuroprotective role in particular to investigate the efficiency in the removal of senescent cells from the tissue.

Taken together, the results prompt us to propose a model of how increased IOP leads to the destruction of retinal structures during glaucoma progression (Figure 5e). During early stages, elevated IOP induces cell‐intrinsic changes leading to cellular senescence and production of SASP. As the disease worsens, SASP molecules induce apoptosis and senescence in neighboring cells. Such changes are largely independent of whether the initial insult is still present or has been eliminated with IOP‐lowering treatment. RGC apoptosis inevitably leads to the loss of axons and optic nerve degeneration. Conversely, when senescent cells (induced directly by elevated IOP) are removed using senolytic drugs (Figure 5e, *bottom*), neighboring cells are not exposed to detrimental SASP and remain healthy. We propose that such treatment can lead reduce the rate of glaucoma worsening. Moreover, we speculate that changes in combination with IOP‐lowering treatments may have even better protection than either type of therapy alone.

We and others previously used 90 mm Hg as an extremely acute and reproducible way to induce cell response and RGC death (Skowronska‐Krawczyk et al., 2015). This level of pressure is likely an ischemic insult. The resulting synchronized and quick cell death provide unique qualities that are extremely useful for molecular and biochemical studies; however such an acute and high‐pressure change is not fully representative of POAG, a chronic optic neuropathy. However, this acute insult allows the study of stress response time course and can help unravel important aspects of stepwise response of retinal cells to elevated IOP which is a daunting task to assess in chronic models the disease. Importantly, our previous data showing the presence of senescent cells in human glaucomatous retinas (Skowronska‐Krawczyk et al., 2015) should stimulate the use of senolytic drugs in other animal models of glaucoma.

Dasatinib is a selective tyrosine kinase receptor inhibitor that is commonly used in the therapy of chronic myelogenous leukemia (CML). Other studies have shown that treatment with dasatinib is effective in destroying senescent fat cell precursors (Zhu et al., 2015). Our RNA‐seq data pointed to this senolytic drug as a potential candidate for in vivo treatment of retinal damage induced by IOP elevation. Notably, we found that the level of RGC protection resembles the one obtained with GCV treatment of p16‐3MR transgenic line. Based on these findings, we conclude that dasatinib treatment resulted in RGC protection through removal of senescent cells. It will be of interest to further investigate the possible therapeutic effects of other senolytic drugs in glaucoma and glaucoma models.

The gene encoding *p16INK4a*, *CDKN2A*, lies within the INK4/ARF tumor suppressor locus on human chromosome 9p21; this is the most significant region to be identified as having an association with POAG in different population samples (Ng, Casson, Burdon, & Craig, 2014). Although the molecular mechanism of many of these associations is yet to be described, we have shown that one of them especially highly correlates with the presence of another top risk variant of glaucoma—Six6 rs33912345. Our study further showed that upregulation of homozygous *SIX6* risk alleles (CC) leads to an increase in *p16Inka* expression, with subsequent cellular senescence (Skowronska‐Krawczyk et al., 2015). Interestingly, others have described an alternative mechanism whereby IOP‐induced TBK1 expression caused an increase of *p16Ink4a* expression through the Akt‐ Bmi1 phosphorylation pathway (Li et al., 2017). Given the complexity of the 9p21 locus, we believe that there are more pathways involved in *p16Ink4a* regulation and further work is needed to understand the role of p16Ink4a as a integrator of these signals especially upon IOP elevation.

Several collaborative efforts identified numerous SNPs localized within the 9p21 locus to be highly associated with the risk of open‐angle glaucoma including normal‐tension glaucoma (NTG), a glaucomatous optic neuropathy not associated with elevated IOP (Killer & Pircher, 2018; Wiggs & Pasquale, 2017). Intriguingly, one of the top variants associated with the risk of NTG is located in the gene TBK1, a factor that has been recently shown to be implicated in upregulation of *p16ink4a* gene (Li et al., 2017). Finally, recent studies have also revealed that specific methylation patterns in the 9p21 locus are strongly associated with the risk of NTG glaucoma (Burdon, 2018). It is notable that the positions of most, if not all, of these SNPs and methylation markers overlap with active regulatory regions within the locus identified by ENCODE (Consortium, 2012). Although regulation of the 9p21 locus in the context of many diseases and aging is under extensive investigation, it still remains to be explicitly addressed in relation to glaucoma.

Another major type of glaucomatous optic neuropathy is angle closure glaucoma (ACG), a condition characterized by blockage of the drainage angle of the eye. To date, there is no study reporting genetic variants or methylation markers in the 9p21 locus significantly associated with the risk of ACG despite several studies implicating various molecular mechanisms (Evangelho, Mogilevskaya, Losada‐Barragan, & Vargas‐Sanchez, 2019). Nevertheless, the fact that progressive vision loss is observed in PACG patients, even after lowering the IOP (Brubaker, 1996), raises the question whether an association could be observed between 9p21 markers and the *progression* rather than the *risk* of the disease. Further studies to unravel such associations are necessary.

Markers of cellular senescence such as expression of the *p16Ink4a* and SASP molecules dramatically increase during aging in both humans and mice. Several studies suggest that *p16Ink4a* + cells act to shorten healthy lifespan by promoting age‐dependent changes that functionally impair tissues and organs (Baker et al., 2016; Childs et al., 2016; Jeon et al., 2017; Krishnamurthy et al., 2006). Intriguingly, a recent explosion of studies has shown that removal of senescent cells using senolytic drugs in progeroid (accelerated aging phenotype) and healthy mice induces lifespan extension and improves the health of animals (Baker et al., 2011, 2016; Scudellari, 2017; Xu et al., 2018). Our studies suggest a potential use of such therapy to reduce glaucoma associated blindness, either as a stand‐alone treatment or together with IOP‐lowering therapies.

## CONFLICT OF INTEREST

Nothing to declare.

## Data Availability

The data that support the findings of this study are openly available in GEO database (GSE141725) and in Dryad at https://doi.org/10.6075/J0707ZTM (Rocha, 2019).
