# Supplementary material for: Early removal of senescent cells protects retinal ganglion cells loss in experimental ocular hypertension
Source: Aging Cell. 2019 Dec 22;19(2):e13089. doi: 10.1111/acel.13089 (PMC6996954; doi:10.1111/acel.13089)
Supplement: Supplementary file 1 [file ACEL-19-e13089-s001.pdf]

kinase perturbation through Enrichr

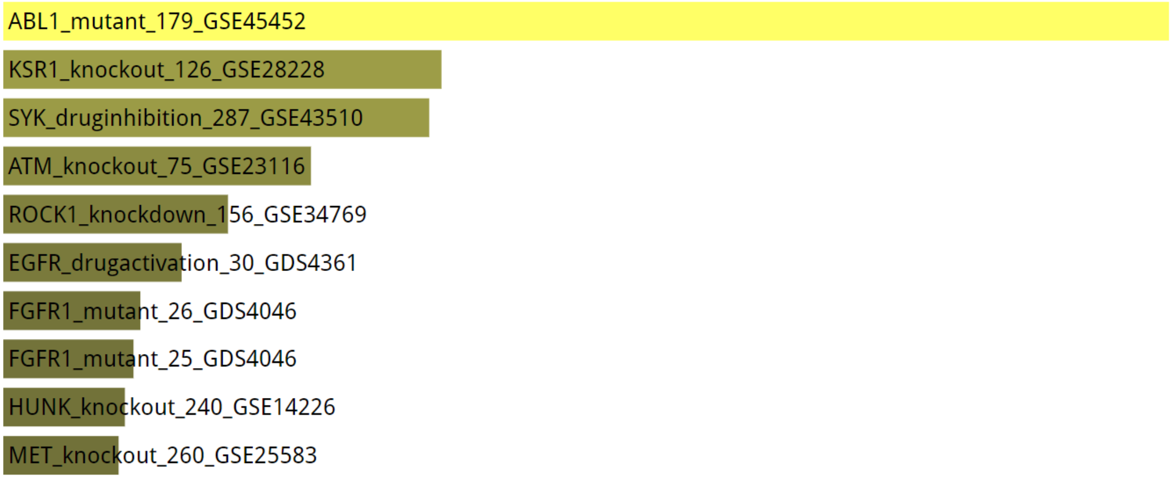

Index	Name	P-value	Adjusted p-value	Z-score	Combined score
1	ABL1_mutant_179_GSE45452	6.591e-11	1.879e-8	-1.78	41.66
2	KSR1_knockout_126_GSE28228	0.000007324	0.0006958	-1.71	20.20
3	SYK_druginhibition_287_GSE43510	0.000007324	0.0006958	-1.68	19.84
4	ATM_knockout_75_GSE23116	0.00006225	0.004435	-1.69	16.35
5	ROCK1_knockdown_156_GSE34769	0.0001696	0.009669	-1.60	13.90
6	EGFR_drugactivation_30_GDS4361	0.0004411	0.02095	-1.62	12.54
7	FGFR1_mutant_26_GDS4046	0.001093	0.02595	-1.66	11.32
8	FGFR1_mutant_25_GDS4046	0.001093	0.02595	-1.63	11.12
9	HUNK_knockout_240_GSE14226	0.001093	0.02595	-1.59	10.86
10	MET_knockout_260_GSE25583	0.001093	0.02595	-1.57	10.68

Supplementary figure 1. Enrichr analysis of genes 617 genes altered in nGCV conditions by IOP.

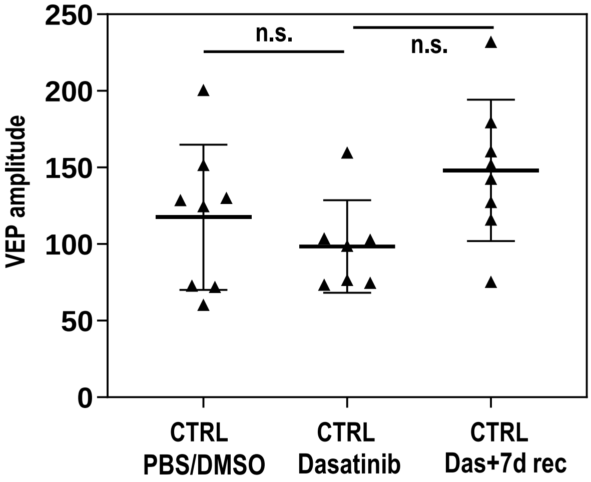

Supplementary figure 2. *Comparison of the healthy eye VEP amplitude. No significant differences detected. N>4 animals in each group . Statistical tests were performed using ANOVA with post-hoc Tukey correction for multiple testing. n.s. – not significant*
